# State of the Art and Uses for the Biopharmaceutics Drug Disposition Classification System (BDDCS): New Additions, Revisions, and Citation References

**DOI:** 10.1208/s12248-022-00687-0

**Published:** 2022-02-23

**Authors:** Giovanni Bocci, Tudor I. Oprea, Leslie Z. Benet

**Affiliations:** 1 Department of Bioengineering and Therapeutic Sciences, Schools of Pharmacy and Medicine, University of California, San Francisco, California, 94143-0912 United States of America; 2 Translational Informatics Division, Department of Internal Medicine, University of New Mexico, Albuquerque, New Mexico 87131 United States of America; 3 ExScientia, The Schrödinger Building, Oxford Science Park, Oxford, OX4 4GE UK; 4 UNM Comprehensive Cancer Center, Albuquerque, New Mexico 87131 United States of America; 5 Department of Rheumatology and Inflammation Research, Institute of Medicine, Sahlgrenska Academy at Gothenburg University, Gothenburg, Sweden; 6 Novo Nordisk Foundation Center for Protein Research, Faculty of Health and Medical Sciences, University of Copenhagen, Copenhagen, Denmark; 7 Roivant Discovery, 451 D Street, Boston, MA 02210 USA

**Keywords:** BDDCS, BCS, DILI, dose number, extent of metabolism, food effects, solubility

## Abstract

The Biopharmaceutics Drug Disposition Classification system (BDDCS) is a four-class approach based on water solubility and extent of metabolism/permeability rate. Based on the BDDCS class to which a drug is assigned, it is possible to predict the role of metabolic enzymes and transporters on the drug disposition of a new molecular entity (NME) prior to its administration to animals or humans. Here, we report a total of 1475 drugs and active metabolites to which the BDDCS is applied. Of these, 379 are new entries, and 1096 are revisions of former classification studies with the addition of references for the approved maximum dose strength, extent of the systemically available drug excreted unchanged in the urine, and lowest solubility over the pH range 1.0–6.8 when such information is available in the literature. We detail revised class assignments of previously misclassified drugs and the literature analyses to classify new drugs. We review the process of solubility assessment for NMEs prior to drug dosing in humans and approved dose classification, as well as the comparison of Biopharmaceutics Classification System (BCS) *versus* BDDCS assignment. We detail the uses of BDDCS in predicting, prior to dosing animals or humans, disposition characteristics, potential brain penetration, food effect, and drug-induced liver injury (DILI) potential. This work provides an update on the current status of the BDDCS and its uses in the drug development process.

**Graphical abstract:**

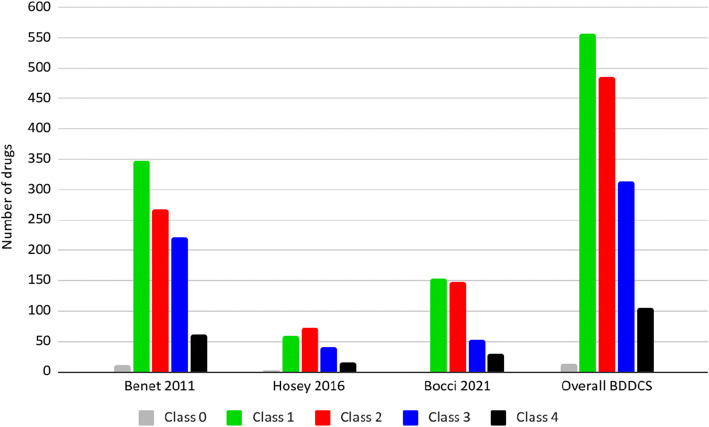

**Supplementary Information:**

The online version contains supplementary material available at 10.1208/s12248-022-00687-0.

## INTRODUCTION

The Biopharmaceutics Drug Disposition Classification System (BDDCS), based on rate of membrane permeability/extent of metabolism and solubility characteristics, was proposed by Wu and Benet (1) as a methodology to predict drug disposition properties. This manuscript reports the BDDCS class for many newly approved drugs and revisits previously published BDDCS collection articles, providing citation references for reported parameters and, in some cases, correcting the classifications previously reported. We update the solubility criterion that has been proposed for the early classification of drugs prior to determination of the human dose; compare Biopharmaceutics Classification System (BCS) assignments with BDDCS assignments when the former are available; and review uses/insights that BDDCS classification provides in early drug development before a new molecular entity (NME) is dosed to animals or humans.

## THE DIFFERENCES BETWEEN BCS AND BDDCS ASSIGNMENT

The BCS, proposed by Amidon *et al.* (2), was developed to reduce the burden of conducting *in vivo* human studies related to regulatory approval and development of new formulations of immediate-release products. Drugs are classified in BCS based on the extent of permeability and the solubility of the active species present in an approved drug product (3). Drugs for which the extent of absorption is greater than 85% (high extent of permeability) are designated as BCS class 1 or 2, while drugs not achieving a high extent of permeability are designated as BCS classes 3 and 4. Further separation is based on measured solubility depending on the dose number (DN). In BDDCS, this parameter is calculated based on the previous FDA criteria of the approved maximum dose strength (MDS), which is the highest approved dose of the drug in milligrams, the drug water solubility as defined by the FDA criterion (SOL_FDA_), which is the lowest drug water solubility (mg/mL) measured across the pH range 1–6.8 and the assumed human gastric volume of 250 mL. These three parameters are necessary to calculate the DN (DN=$\frac{MDS}{SOL_{FDA}\bullet 250}\Big)$. BDDCS classification utilizes the same DN characteristics as BCS for approved drugs prior to May 2021 to differentiate classes 1 and 3 (high solubility) from classes 2 and 4 (low solubility). The updated BCS regulations harmonized through ICH (3) now defines solubility in terms of the highest single therapeutic dose. The effect of this difference will be discussed in a subsequent section.

However, Wu and Benet (1) recognized that the rate of intestinal permeability (rather than the extent) could lead to the prediction of the extent of metabolism (EoM) of a drug. The high intestinal permeability rate is the defining characteristic of BDDCS classes 1 and 2 drugs, while low intestinal permeability rate is the defining characteristic for BDDCS classes 3 and 4 drugs. Passive drug membrane permeability rate in any relevant membrane such as a Caco-2 cell line or even a nonbiologic PAMPA (4) provides a reasonable estimate of EoM. Wu and Benet (1) reported that the vast majority of approved drugs were either EoM ≥ 70% or EoM ≤ 30%, easily separating BDDCS classes 1 and 2 drugs from classes 3 and 4 drugs. The fraction of the available dose that is excreted unchanged in urine (*f*_*e*_) can be translated into a measurement of a drug’s EoM. Drugs exhibiting *f*_*e*_ values ≤ 30% were considered extensively metabolized, high permeability BDDCS classes 1 and 2. This estimate could be confounded by marked biliary elimination of unchanged drug, but information concerning a drug’s metabolic elimination and potential biliary elimination was considered in making the BDDCS assignment.

Wu and Benet (1) further reasoned that poor passive permeability drugs (BDDCS classes 3 and 4) would require transporters to achieve membrane permeability, but that transporters may not significantly affect drug disposition for high permeability rate drugs, especially for highly soluble BDDCS class 1 drugs where high concentrations of drug would be available for passive diffusion. Therefore, although the high permeability rate BDDCS class 2 drugs are primarily metabolized, transporters may or may not be clinically relevant in drug disposition due to the lower available concentration resulting from their low solubility characteristics.

## NEW ADDITIONS TO BDDCS

**Fig. 1:**
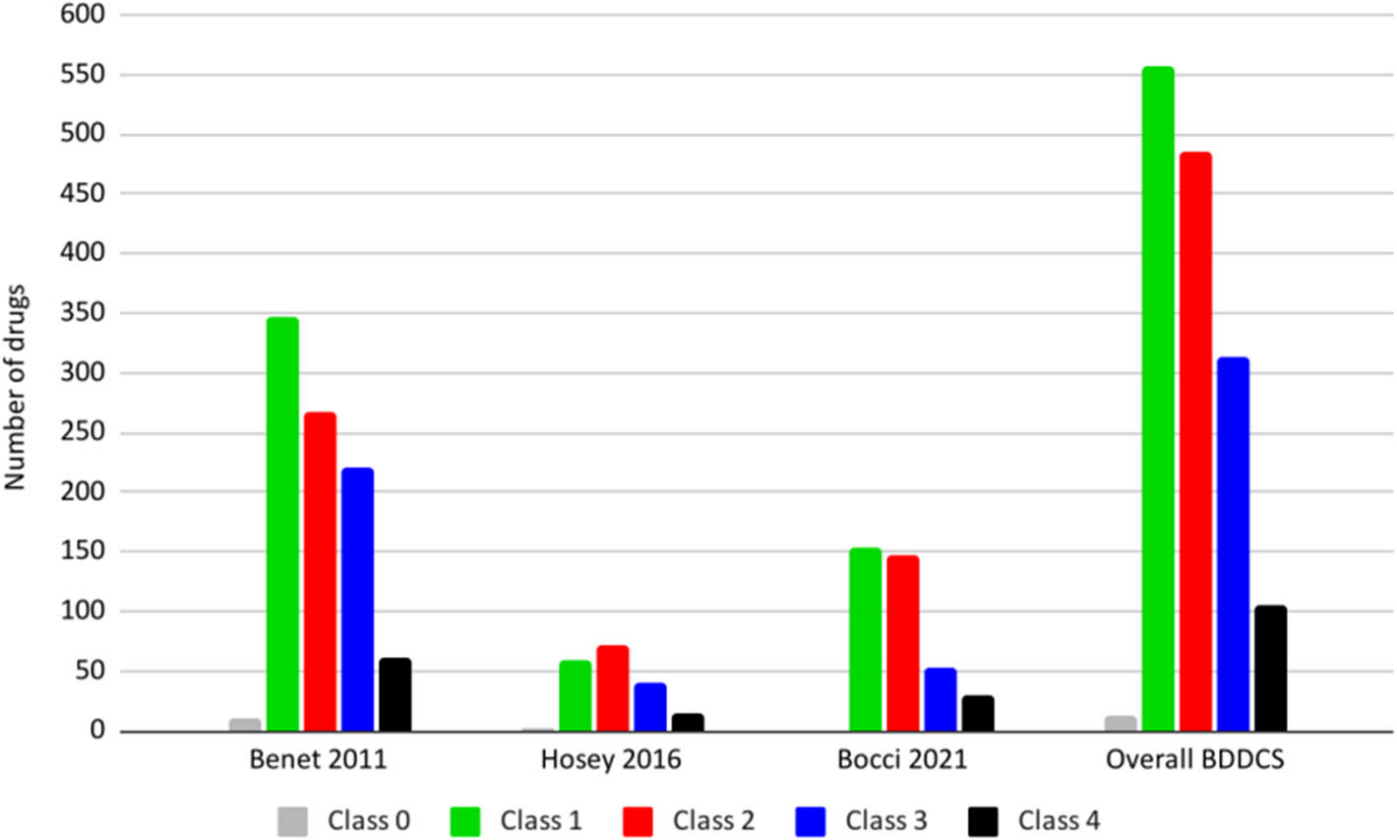
State of the art for the drugs classified with the BDDCS across all collections over time

**Fig. 2:**
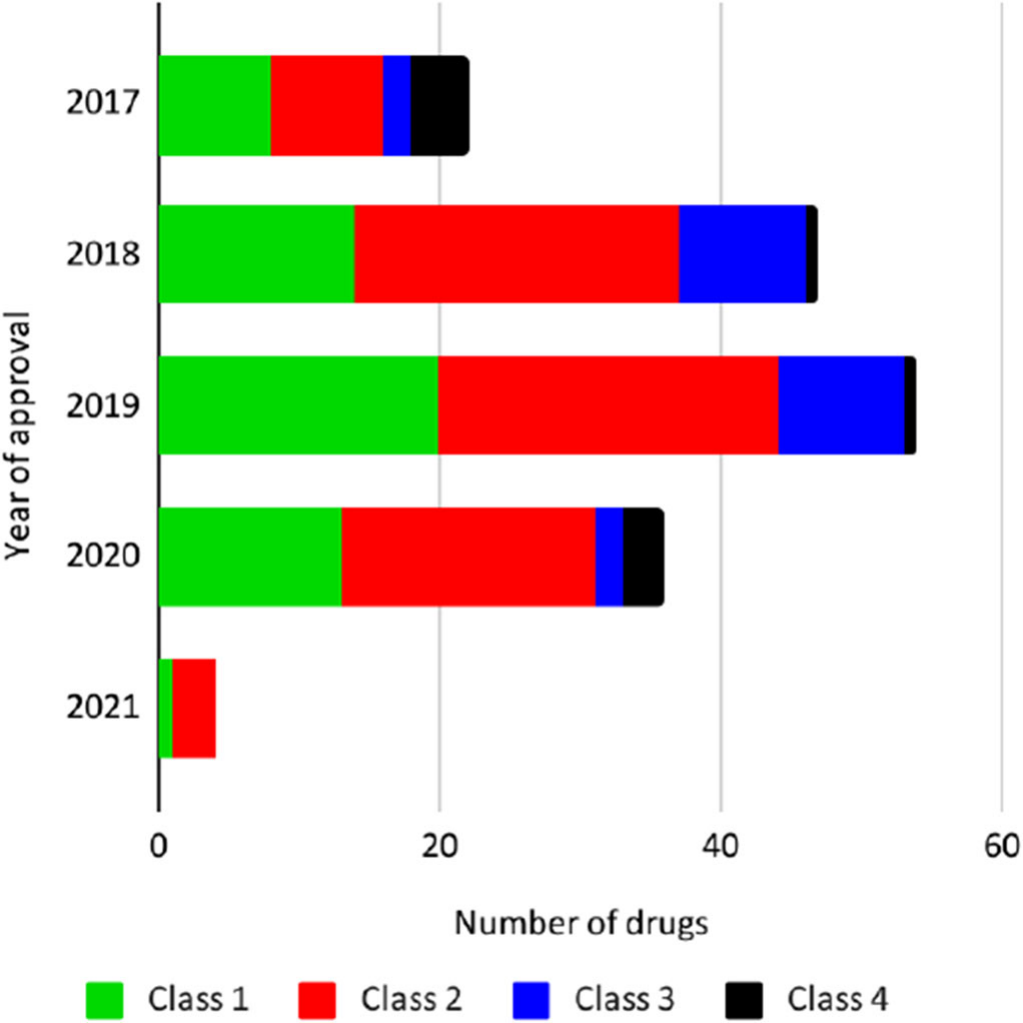
BDDCS classes distribution over recent years

Although the BDDCS was first introduced in 2005 (1), the two major works listing drugs and their BDDCS class are the 2011 paper of Benet *et al.*(5) and the 2016 paper by Hosey *et al.*(6). Since then, no further multiple BDDCS classifications were provided to the scientific community. Our work here aims to provide new BDDCS assignments for drugs not previously listed. We compiled a list of 140 drugs approved between 2017 and 2020 enriched with older drugs that were not previously classified for a final number of 379 newly classified drugs. We carefully inspected the literature to retrieve the information necessary for assessing the BDDCS class of these 379 additional drugs and reviewed the previously listed 1096 assignments. The results of these new assignments are depicted in Fig. 1, together with the previous classification of Benet *et al.* (5) and Hosey *et al.* (6) and the distribution of the total 1475 compounds. For a few drugs, the value of *f*_*e*_ can be susceptible to urine pH changes, so much so that classification can change from classes 1 and 3 to classes 2 and 4 depending on urine pH. These drugs are listed as BDDCS class 0. For the previously 379 BDDCS unclassified drugs, we report 151 class 1, 147 class 2, 52 class 3, and 29 class 4 drugs. The list of these newly classified BDDCS drugs can be found in Table I and in Supporting information. The distribution of BDDCS class for newly approved drugs since 2017 in our analysis is presented in Fig. 2, demonstrating the predominance of class 2, followed by class 1.

**Table I:** New BDDCS classifications

Name	BDDCS
Abametapir	2
Abemaciclib	1
Abiraterone	4
Abiraterone acetate	2
Acalabrutinib	2
Acemetacin	2
Acenocoumarol	2
Acetylcholine chloride	1
Acetylmethadol	1
Adefovir	3
Ademetionine butane disulfonate	1
Adinazolam	2
Afamelanotide acetate	1
Alatrofloxacin mesylate	1
Alectinib hydrochloride	2
Alimemazine tartrate	3
Alizapride hydrochloride	3
Alogliptin	3
Alpelisib	2
Ambenonium chloride	3
Amifampridine phosphate	1
Aminolevulinic acid hydrochloride	1
Amobarbital sodium	1
Amodiaquine	2
Anagrelide hydrochloride	2
Antazoline mesylate	1
Antofloxacin hydrochloride	3
Apalutamide	2
Apixaban	1
Arbekacin	3
Arbutin	1
Arsenic trioxide	1
Artemisinin	2
Artesunate	2
Atrasentan hydrochloride	1
Aurothioglucose hydrate	3
Avapritinib	2
Avatrombopag maleate	2
Avibactam	3
Azatadine	1
Bacampicillin hydrochloride	1
Baloxavir	2
Baloxavir marboxil	2
Balsalazide disodium	1
Baricitinib	3
Beclomethasone dipropionate	2
Bempedoic acid	2
Benfluorex hydrochloride	1
Benzthiazide	4
Berotralstat hydrochloride	1
Betahistine dihydrochloride	1
Betrixaban maleate	1
Bictegravir sodium	2
Binimetinib	1
Bisacodyl	2
Bleomycin sulfate	3
Brexanolone	2
Brigatinib	2
Brivaracetam	1
Brivudine	2
Bromopride	2
Brompheniramine maleate	1
Bunazosin	1
Buserelin acetate	3
Butobarbital sodium	1
Cabazitaxel	2
Calcitonin (salmon synthetic)	1
Camylofine dihydrochloride	2
Cangrelor tetrasodium	1
Cannabidiol	2
Capmatinib hydrochloride	2
Carboprost tromethamine	1
Carglumic acid	1
Cedazuridine	1
Cefcanel daloxate hydrochloride	1
Cefetamet	4
Cefetamet pivoxil	2
Cefiderocol sulfate tosylate	3
Cefozopran hydrochloride	3
Cenobamate	1
Cholestyramine	2
Cibenzoline	3
Clascoterone	2
Clemizole hydrochloride	1
Clenbuterol hydrochloride	1
Clobetasol propionate	2
Cobimetinib fumarate	1
Colestipol	4
Copanlisib dihydrochloride	1
Crisaborole	2
Cyclothiazide	3
Dacomitinib	2
Dapoxetine hydrochloride	1
Darolutamide	2
Decitabine	1
Deferoxamine mesylate	1
Deflazacort	2
Delafloxacin meglumine	4
Deutetrabenazine	1
Dexbrompheniramine	1
Dexchlorpheniramine	1
Dexlansoprazole	2
Dexmedetomidine hydrochloride	1
Dichloroacetic acid	1
Dichlorphenamide	4
Dicyclomine hydrochloride	1
Diethylpropion hydrochloride	1
Dihydrocodeine bitartrate	1
Dihydrodydrogesterone	4
Diphenoxylate hydrochloride	2
Dirithromycin	4
Doravirine	2
Doxacurium chloride	4
Doxapram hydrochloride	1
Doxylamine succinate	3
Droperidol	2
Drotaverine	1
Droxidopa	1
Duvelisib	2
Dydrogesterone	2
Dyphylline	3
Econazole nitrate	2
Edaravone	2
Elagolix sodium	1
Elbasvir	4
Elexacaftor	1
Enasidenib mesylate	2
Encorafenib	2
Enoximone	2
Entrectinib	2
Ephedrine	3
Epinastine hydrochloride	3
Epinephrine	1
Eravacycline dihydrochloride	1
Erdafitinib	2
Eribulin mesylate	3
Ertugliflozin L-pyroglutamic acid	1
Eslicarbazepine	3
Estramustine	2
Estramustine phosphate	1
Estriol	1
Etelcalcetide hydrochloride	3
Ethacrynic acid	4
Ethionamide	2
Ethoxzolamide	4
Ethylene glycol	1
Ethynodiol diacetate	1
Etofibrate	2
Etretinate	2
Favipiravir	1
Fedratinib dihydrochloride	2
Fenoldopam mesylate	2
Ferric maltol	1
Floxuridine	3
Fluorescein sodium	1
Fominoben	1
Fondaparinux	3
Fosnetupitant chloride hydrochloride	1
Fosphenytoin sodium	1
Fospropofol disodium	1
Fostamatinib disodium hexahydrate	2
Fostemsavir tromethamine	1
Furamidine	4
Furazolidone	2
Gabapentin enacarbil	2
Gabexate mesylate	1
Gadofosveset trisodium	3
Gadoteridol	3
Gamma hydroxybutyric acid	1
Garenoxacin mesylate	3
Gatifloxacin	3
Gemifloxacin mesylate	4
Gilteritinib fumarate	2
Givosiran	1
Glasdegib maleate	1
Glecaprevir	4
Glucose	1
Glutethimide	2
Glycerol	1
Glycerol phenylbutyrate	2
Grazoprevir	4
Guaifenesin	1
Halofantrine	2
Ibrexafungerp	2
Infigratinib	2
Irofulven	2
Isomazole	1
Isoxicam	2
Istradefylline	2
Ivosidenib	2
Ixazomib	1
Ketobemidone	1
Lactitol	1
Lactose	1
Lactulose	1
Larotrectinib sulfate	1
Lasmiditan hemisuccinate	1
Lefamulin acetate	1
Lemborexant	2
Lercanidipine hydrochloride	2
Letermovir	4
Levocarnitine	1
Levoleucovorin	1
Levomethadyl acetate hydrochloride	1
Levorphanol tartrate	1
Linagliptin	3
Lindane	2
Lomustine	2
Lonafarnib	2
Lorlatinib	2
Lormetazepam	2
Loxapine succinate	1
Lubiprostone	2
Lumateperone tosylate	1
Lurbinectedin	2
Lusutrombopag	1
Macitentan	2
Mannitol	3
Mazindol	1
Melagatran	4
Melperone	1
Mepenzolate	1
Metazosin	4
Methacycline	3
Methionine	1
Methsuximide	1
Methylparaben	1
Methyltestosterone	2
Meticrane	4
Metildigoxin	3
Mevastatin	2
Midodrine hydrochloride	1
Midostaurin	2
Migalastat hydrochloride	3
Mitiglinide	1
Mitomycin	1
Mitotane	2
Mizoribine	3
Moclobemide	1
Moexipril hydrochloride	1
Moexiprilat	3
Moxidectin	2
Moxonidine	4
Nabilone	2
Naftopidil	2
Naldemedine tosylate	1
Nandrolone	1
Nandrolone decanoate	2
Nebivolol hydrochloride	2
Neratinib maleate	2
Netarsudil dimesylate	1
Netupitant	2
Niraparib tosylate	1
Nitazoxanide	2
Noradrenaline	1
Obeticholic acid	2
Oliceridine fumarate	1
Olsalazine sodium	1
Omadacycline tosylate	3
Opicapone	2
Osilodrostat phosphate	1
Oxyphenbutazone	2
Oxyphenonium bromide	1
Oxytocin	1
Ozanimod hydrochloride	1
Pafuramidine	2
Papaverine hydrochloride	1
Pegaptanib sodium	1
Pemigatinib	2
Pentachlorophenol	2
Perphenazine	2
Pexidartinib hydrochloride	2
Phenelzine sulfate	1
Pheniramine maleate	1
Phenol	1
Phenprocoumon	1
Pibrentasvir	4
Pidotimod	3
Pilsicainide hydrochloride	3
Pimavanserin tartrate	1
Pinaverium bromide	1
Piperacetazine	1
Pipobroman	1
Pitolisant hydrochloride	1
Plazomicin sulfate	3
Plecanatide	1
Polythiazide	2
Pralsetinib	2
Pranlukast	2
Pregnenolone	2
Pretomanid	2
Pridinol	2
Procarbazine hydrochloride	1
Propiverine hydrochloride	1
Propylparaben	2
Prucalopride succinate	3
Rasagiline mesylate	1
Recainam	3
Relebactam	3
Relugolix	2
Remdesivir	2
Remimazolam besylate	1
Remoxipride hydrochloride	1
Revefenacin	1
Ribociclib succinate	1
Rifamycin sodium	3
Rifapentine	2
Rimegepant sulfate	4
Ripretinib	2
Risdiplam	2
Rivaroxaban	2
Rucaparib camsylate	2
Safinamide mesylate	1
Samidorphan	1
Sarecycline hydrochloride	3
Secnidazole	1
Selinexor	2
Selpercatinib	1
Selumetinib sulfate	1
Semaglutide	2
Sematilide hydrochloride	3
Semaxanib	2
Setmelanotide acetate	3
Sevelamer	4
Silodosin	2
Siponimod fumarate	2
Sitaxentan sodium	1
Solriamfetol hydrochloride	3
Sorbitol	1
Sorivudine	2
Sotorasib	2
Stanozolol	1
Stiripentol	1
Succimer	1
Sulfaphenazole	2
Tafamidis	2
Tafamidis meglumine	2
Tafenoquine succinate	2
Talazoparib tosylate	3
Tapentadol hydrochloride	1
Tazemetostat hydrobromide	2
Tecovirimat	2
Tedizolid	2
Telbivudine	3
Telotristat	2
Telotristat ethyl etiprate	2
Temsavir	2
Tenapanor hydrochloride	2
Tenofovir	3
Tenofovir alafenamide fumarate	1
Tezacaftor	2
Theobromine	1
Tizoxanide	4
Tranilast	2
Trichlormethiazide	3
Triclosan	2
Trifarotene	2
Triheptanoin	2
Trimetaphan	2
Trimethobenzamide hydrochloride	2
Tripelennamine hydrochloride	1
Triprolidine hydrochloride	1
Troleandomycin	2
Tucatinib	2
Ubrogepant	2
Upadacitinib	1
Uracil mustard	1
Vaborbactam	4
Valbenazine ditosylate	2
Valpromide	2
Velpatasvir	4
Venetoclax	2
Vibegron	4
Vildagliptin	1
Viloxazine hydrochloride	1
Voxelotor	2
Voxilaprevir	4
Ximelagatran	2
Zanubrutinib	2
Zimeldine	1
Zuclopenthixol dihydrochloride	1

For the drugs lusutrombopag and binimetinib, a precise class could not be assigned. These two drugs are extensively metabolized, but we could not find any information regarding their solubility. However, no food effects are reported in their labels, which suggest that their classification is BDDCS 1 drugs (1, 7).

## BDDCS REVISIONS

Hosey and co-workers (6) identified some drugs that had been previously misclassified either because their EoM was wrongly annotated or because biliary excretion was not considered, when it was the predominant elimination route for the unchanged drug. Upon applying these corrections, drugs were correctly reclassified to different BDDCS classes (6). Here, we extend the revision work to the 1096 drugs reported previously (1, 5) by reviewing EoM and SOL_FDA_ data reporting each value and the reference(s) with supporting data. We also made a number of BDDCS classification revisions. In Table II, we summarize the results of the BDDCS revision work.

**Table II:**
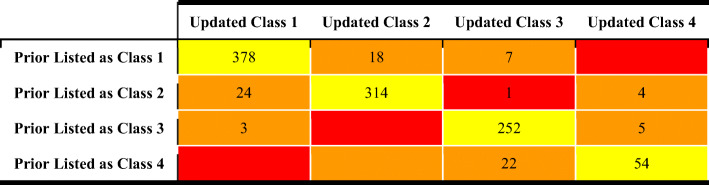
BDDCS class changes from former publications. The number of drugs with: BDDCS class unchanged (yellow), single property BDDCS class change (orange), double property BDDCS class change (red)

The great majority of the reviewed drugs (92.2%) retain their former BDDCS assigned class. Of the drugs that had a class change, the vast majority had a single property class change, which means that either the EoM or the SOL_FDA_ updated value caused the change in class. For example, 18 BDDCS class 1 and five BDDCS class 3 drugs were found to have a low solubility in the literature. Since their EoM was confirmed, these drugs were reassigned to either BDDCS class 2 or 4, respectively. Alternatively, the solubilities of 24 BDDCS class 2 and 22 BDDCS class 4 drugs were, instead, found to be high, while their EoMs were substantially confirmed. Thus, 24 previously listed BDDCS class 2 drugs were reassigned as class 1, and 22 class 4 drugs were reassigned as class 3. Furthermore, the EoM values for seven BDDCS class 1 and four BDDCS class 2 drugs were found to be low in the literature, with no critical changes in solubility. Hence, these drugs were reassigned as BDDCS class 3 and class 4, respectively. Moreover, where solubility values were confirmed, there were a few cases where the opposite class change occurred. Similarly, three BDDCS class 3 drugs were re-classified as BDDCS class 1 because their EoM were high. However, we do not report any class change from BDDCS class 4 to BDDCS class 2. The only drug for which we detected a double property class change is fialuridine, which is revised from BDDCS class 2 to BDDCS class 3. The complete list of 84 drugs for which we report a change of BDDCS class is in Table III. Complete revision details are provided in Supporting information Table S3.

**Table III:** BDDCS class changes from initial publications

Name	Prior listed BDDCS	Corrected BDDCS	Initial publication
Acarbose	1	3	(5)
Adefovir dipivoxil	3	1	(5)
Alpidem	1	2	(6)
Amifloxacin	3	4	(6)
Amineptine	1	2	(6)
Artemether	2	1	(5)
Azithromycin dihydrate	3	4	(5)
Bendazac lysine	2	1	(6)
Betamipron	3	4	(5)
Bethanechol chloride	3	1	(6)
Candesartan cilexetil	4	2	(5)
Carbovir	4	3	(6)
Cefadroxil	3	4	(5)
Cefmetazole sodium	3	4	(5)
Ceftazidime	3	4	(5)
Chlorhexidine gluconate	3	4	(6)
Cladribine	2	1	(5)
Clinafloxacin	3	4	(6)
Clodronic acid	4	3	(5)
Daclatasvir dihydrochloride	4	3	(6)
Daunorubicin	2	1	(5)
Dexloxiglumide	1	2	(6)
Dihydroergotamine mesylate	1	2	(6)
Ergotamine tartrate	1	2	(5)
Etoposide	3	4	(5)
Everolimus	1	2	(5)
Fialuridine	2	3	(6)
Finasteride	1	2	(5)
Fipexide	3	2	(6)
Flavoxate hydrochloride	2	1	(6)
Fluticasone propionate	2	1	(5)
Fusidic acid sodium	2	1	(6)
Genistein	1	2	(6)
Guanethidine sulfate	1	3	(6)
Lenalidomide	4	3	(5)
Levonorgestrel	4	2	(5)
Licarbazepine acetate	1	2	(6)
Liothyronine sodium	2	1	(6)
Loperamide hydrochloride	3	1	(5)
Medroxyprogesterone acetate	4	2	(5)
Megestrol acetate	4	2	(5)
Melphalan hydrochloride	1	2	(5)
Mephenytoin	2	1	(6)
Methylprednisolone	1	2	(5)
Meticillin	3	4	(5)
Metolazone	3	4	(6)
Metyrapone	1	2	(6)
Metyrosine	4	3	(6)
Mibefradil dihydrochloride	2	1	(6)
Milrinone	3	4	(5)
Nystatin	3	4	(5)
Omeprazole	1	2	(5)
Oxymetholone	1	2	(6)
Oxytetracycline	3	4	(6)
P-aminosalicylic acid	1	2	(5)
Pancuronium bromide	3	4	(5)
Penbutolol	2	1	(6)
Phenylethylmalonamide	3	4	(5)
Practolol	3	4	(6)
Procainamide hydrochloride	3	4	(5)
Prochlorperazine	1	2	(5)
Pyrimethamine	3	1	(5)
Quinapril hydrochloride	2	1	(5)
Raltegravir potassium	2	1	(5)
Regadenoson	3	4	(5)
Repaglinide	2	1	(5)
Ritodrine	3	1	(5)
Roquinimex	2	1	(6)
Sofosbuvir	3	1	(6)
Sparfloxacin	1	2	(5)
Talinolol	3	4	(5)
Tedizolid phosphate	1	2	(6)
Telithromycin	2	1	(5)
Temafloxacin hydrochloride	3	4	(6)
Temocapril hydrochloride	1	2	(5)
Temocaprilat	3	4	(5)
Temozolomide	2	1	(5)
Temsirolimus	1	2	(5)
Tenofovir disoproxil fumarate	3	1	(5)
Tetrabenazine	2	1	(5)
Thioridazine	1	2	(5)
Tizanidine hydrochloride	2	1	(5)
Triamcinolone acetonide	1	2	(5)
Trovafloxacin mesylate	1	3	(6)
Verapamil hydrochloride	1	2	(5)
Zaleplon	2	1	(5)

## DISCREPANCIES BETWEEN BDDCS AND BCS PREDICTIONS

Major drug regulatory agencies use the BCS (2) to assess the eligibility of drugs for a waiver of *in vivo* bioequivalence studies (3, 8). In other words, two drug products containing the same drug substance can be considered bioequivalent if their rate and extent of availability (after oral administration, at the same molar dose) lie within acceptable predefined limits. BCS classes 1 (high solubility, high permeability) and 3 (high solubility, low permeability) immediate-release orally dosed drugs are eligible for biowaivers. The list of the 257 drugs for which we could determine both BCS and BDDCS classification is in Table IV. Almost all of these 257 drugs were assigned their BCS class based on the previous MDS solubility criterion, not the revised highest therapeutic dose criterion (3); therefore in this compilation, we continue to use MDS in the BDDCS classification. We believe this change in BCS criteria will have little if any impact on the usefulness of BDDCS, Because of confidentiality issues, regulatory agencies do not identify the number or the names of specific drugs eligible for a biowaiver, and we have no way of knowing whether these published BCS classifications have been allowed biowaivers, yet since we could only locate a BCS class designation for 257 of the 1475 BDDCS classified drugs detailed here, we believe that regulatory agencies have accepted relatively few drugs to be biowaiver eligible. BCS classes 2 (low solubility, high permeability) and 4 (low solubility, low permeability) drugs are not eligible. The BDDCS was intended to expand the number of BCS classes 1 and 3 drugs eligible for a biowaiver (for drugs not BCS classified) and predict all drugs’ disposition profiles (3). However, as noted by Metry and Polli (9), the harmonized BCS criteria will lead to even fewer drugs eligible for biowaivers.

**Table IV:** The current BCS and BDDCS class for drugs where both assignments are available

Name	BCS	BDDCS
Abacavir sulfate	1|3	1
Acalabrutinib	2	2
Acetaminophen	1|3	1
Acetazolamide	4	3
Acetylsalicylic acid	3	1
Acyclovir sodium	1|3	4
Albendazole	2|4	2
Albuterol sulfate	1	3
Allopurinol	3	2
Alprenolol	1	1
Amantadine hydrochloride	1	3
Amiloride	1|3	3
Amiodarone hydrochloride	2|4	2
Amitriptyline hydrochloride	1|2	1
Amodiaquine	2	2
Amoxicillin	1|3	3
Amphotericin B	4	2
Antipyrine	1	1
Astemizole	2	2
Atenolol	3	3
Atorvastatin calcium	2	2
Atropine sulfate	1|3	3
Azathioprine	4	1
Azithromycin dihydrate	2	4
Baricitinib	3	3
Bendroflumethiazide	2	3
Benznidazole	1	1
Benzthiazide	4	4
Bidisomide	3	3
Biperiden	3	1
Buspirone hydrochloride	1	2
Caffeine	1	1
Captopril	1|3	3
Carbamazepine	2	2
Carvedilol	2	2
Cefazolin sodium	3	3
Cetirizine hydrochloride	3	3
Chloramphenicol	3	1
Chloroquine	1	3
Chlorothiazide sodium	4	4
Chlorpheniramine maleate	1|3	1
Chlorpromazine hydrochloride	2|4	1
Chlorthalidone	4	4
Chlorzoxazone	2	2
Cimetidine	3	3
Ciprofloxacin hydrochloride	2|3|4	4
Cisapride	2	2
Clofazimine	2|4	2
Clomiphene citrate	1|3	1
Clomipramine	1|3	1
Cloxacillin	3	4
Codeine monohydrate	3	1
Colchicine	3	3
Cyclophosphamide	1	1
Cyclosporine	2	2
Dacomitinib	2	2
Danazol	2	2
Dapsone	2	2
Darolutamide	2	2
Desipramine hydrochloride	1	1
Dexamethasone	1|3	1
Diazepam	1	1
Diclofenac sodium	2	2
Dicloxacillin	3	3
Didanosine	3	3
Diethylcarbamazine citrate	1	0
Diflunisal	2	2
Digoxin	1|2	3
Diloxanide furoate	2|4	2
Diltiazem	1	1
Diphenhydramine hydrochloride	1	1
Disopyramide	1	3
Doravirine	2	2
Doxepin hydrochloride	1	1
Doxycycline hyclate	1	3
Duvelisib	4	2
Efavirenz	2|4	2
Elagolix sodium	3	1
Enalapril	1	1
Encorafenib	2	2
Ephedrine	1	3
Erdafitinib	1	2
Ergonovine	1|3	1
Ergotamine tartrate	3	2
Ertugliflozin	1	1
Erythromycin	2|3	3
Erythromycin lactobionate	2|3	3
Erythromycin stearate	2|3	4
Ethambutol hydrochloride	1|3	3
Ethinylestradiol	1|3	1
Ethosuximide	1	1
Famotidine	3	3
Fexofenadine hydrochloride	3	3
Fluconazole	1	3
Flufenamic acid	2	2
Fluoxetine hydrochloride	1	1
Flurbiprofen	2	2
Folic acid	2|4	2
Fosamprenavir calcium	1	2
Furosemide	3|4	4
Ganciclovir sodium	3	3
Gilteritinib	4	2
Glipizide	2	2
Glucose	1	1
Glyburide	2|4	2
Griseofulvin	2	2
Haloperidol	2|4	2
Hydralazine hydrochloride	3	1
Hydrochlorothiazide	3|4	3
Ibuprofen	2	2
Imipramine hydrochloride	1	1
Indinavir sulfate	2|4	2
Indomethacin	2	2
Iopanoic acid	2	4
Isoniazid	1	1
Isosorbide dinitrate	1|3	1
Itraconazole	2	2
Ivermectin	2|4	1
Ivosidenib	2	2
Ketoconazole	2	2
Ketoprofen	1	2
Ketorolac tromethamine	1	3
Labetalol	1	1
Lamivudine	1|3	3
Lansoprazole	2	2
Lemborexant	2	2
Letermovir	2	4
Leucovorin calcium	3	3
Levamisole	1|3	1
Levodopa	1	1
Levofloxacin	1	3
Levonorgestrel	1	2
Lidocaine	1	1
Lisinopril	3	3
Lithium carbonate	1	3
Lomefloxacin	1	3
Loperamide hydrochloride	4	1
Lopinavir	2|4	2
Lovastatin	2	2
Macitentan	2	2
Maprotiline	1	1
Mebendazole	2|4	2
Meclizine hydrochloride	4	1
Meclofenamic acid sodium	2	2
Mefenamic acid	2	2
Mefloquine	2|4	2
Meperidine	1	1
Metformin hydrochloride	3	3
Methionine	1	1
Methotrexate	3|4	3
Methyldopa	3	3
Metoclopramide hydrochloride	1|3	1
Metoprolol tartrate	1	1
Metronidazole	1	1
Miconazole nitrate	4	2
Midazolam hydrochloride	1	1
Minocycline hydrochloride	1	1
Misoprostol	1	1
Morphine hydrochloride	1|3	1
Nadolol	3	3
Nalidixic acid	2	2
Naproxen sodium	2	2
Nelfinavir	2|4	2
Neomycin b sulfate	4	3
Neostigmine methylsulfate	3	3
Netupitant	2	2
Nevirapine	2	2
Niacinamide	1	1
Niclosamide	2|4	4
Nifedipine	1|2	2
Nifurtimox	3	2
Nitrofurantoin	2	4
Nitroglycerin	1|3	1
Norethindrone	1	1
Norfloxacin	4	4
Norgestrel	1	1
Nortriptyline	1	1
Nystatin	3|4	4
Ofloxacin	2	3
Orphenadrine	1	1
Oxaprozin	2	2
Papaverine hydrochloride	2	1
Penicillamine	3	3
Penicillin V	1	4
Phenazopyridine hydrochloride	2	2
Phenobarbital	1	1
Phenylbutazone	2	1
Phenytoin sodium	2	2
Pindolol	1	1
Piroxicam	2	2
Pravastatin sodium	3	3
Praziquantel	2	2
Prednisolone	1	1
Primaquine	1	1
Probenecid	2	2
Prochlorperazine	2	2
Proguanil	1	1
Promazine hydrochloride	1	1
Promethazine hydrochloride	1|3	1
Propranolol hydrochloride	1	1
Propylthiouracil	3	1
Pyrantel pamoate	2|4	2
Pyrazinamide	1	1
Pyridostigmine bromide	3	3
Pyrimethamine	2|4	1
Quinidine sulfate dihydrate	1	1
Quinine bisulfate heptahydrate	1|3	1
Raloxifene	2	2
Ranitidine hydrochloride	3	3
Reserpine	3	1
Ribociclib	4	1
Rifampin	2	2
Risperidone	2	1
Ritonavir	2|4	2
Rosiglitazone maleate	1	2
Salicylic acid	1	1
Saquinavir methanesulfonate	2|4	2
Sarecycline	3	3
Selinexor	2	2
Selumetinib sulfate	4	1
Semaglutide	4	2
Sertraline hydrochloride	2	1
Siponimod	2	2
Sirolimus	2	2
Solriamfetol	1	3
Spironolactone	2|4	2
Stavudine	1	3
Sulfadiazine	2|4	4
Sulfamethoxazole	2	2
Sulfasalazine	2|4	2
Sulindac	2	2
Tacrolimus	2	2
Talinolol	2	4
Tamoxifen	2	1
Terfenadine	2|4	2
Tetracycline hydrochloride	3	3
Theophylline anhydrous	1	1
Thyroxine	3	2
Tolmetin	2	2
Tramadol	1	1
Trichlormethiazide	3	3
Triclabendazole	2|4	2
Trimethoprim	2|3	3
Ubrogepant	4	2
Valproic acid	1|2	1
Valsartan	3	4
Verapamil hydrochloride	1|2	2
Vitamin A	2|4	2
Vitamin B1	3	3
Vitamin B2	1	4
Vitamin B6	1	1
Vitamin C	3	0
Vitamin D2	3	2
Warfarin	1|2	2
Zalcitabine	3	3
Zidovudine	1	1

BCS class assignment is ambiguous in some cases because the permeability assignment relies on absorption measurements in humans that are often uncertain and difficult to perform and the lack of intravenous dosing data. Supporting information Table S5 lists the BCS classification for all drugs with appropriate references. Table V summarizes the agreement between the two classification systems. Classification differences between BCS and BDDCS are caused by two factors. First is the definition of permeability. In BCS, high permeability refers to high extent of absorption (greater than 85%) whereas in BDDCS high permeability refers to a high rate of permeability. Therefore, it is possible that a BCS class 1 drug would be classified as BDDCS class 3 if it has a low permeability rate, but the overall extent of absorption is high. The 13 BCS class 1 drugs in Tables IV and V that are BDDCS class 3 are probably due to this reason. For biowaivers, this difference is not relevant since both BCS classes 1 and 3 drugs are eligible. However, predictions of the importance o\f transporters in the disposition of these drugs are less accurate using the BCS class 1 designation. The second factor leading to differences in BCS and BDDCS assignment is the lesser accuracy of *in vitro* permeability measures in BCS translating to extent of permeability *versus* the accuracy of EoM assessments utilized in BDDCS. As Wu and Benet (1) state, the use of EoM over permeability (i.e., BDDCS over BCS) is preferable because after drug approval, it is easier to quantify EoM than extent of absorption as reflected in the multiple BCS assignments for many drugs as shown in Table IV. As expected, a large fraction of BCS classes 1, 2, and 3 are in agreement with their corresponding BDDCS classes (69%, 81%, and 64% respectively); thus, confirming the somewhat decent correlation between extent of absorption and extent of metabolism of drugs. However, the agreement drops markedly for BCS class 4 drugs, where only 4 out of 17 (23%) are confirmed as BDDCS class 4 drugs. It is worth noting that the BCS class 4 drugs azathioprine, loperamide, meclizine, ribociclib, and selumetinib, utilizing the BDDCS classification based on the solubility values referenced here, would have made them eligible for a biowaiver. This discrepancy emerges from both their high extent of metabolism and from suspected errors in solubility class assignments. From our analysis, these drugs should be classified as BDDCS 1. This difference in the permeability criteria makes it much simpler to assign BDDCS class *versus* BCS class. This observation is supported by the number of drugs currently classified by the two methods (not even 300 for BCS *versus* almost 1500 for BDDCS).

**Table V:**
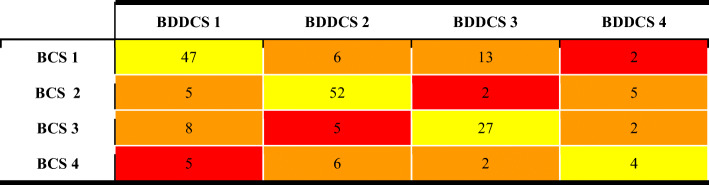
Changes in the classification of drugs when shifting from BCS to BDDCS: no change (yellow), moderate change (orange), complete change (red)

## ASSIGNMENT OF BDDCS CLASS FOR AN NME PRIOR TO *IN VIVO* STUDIES

BCS class assignment can only be made after MDS is established allowing DN to be determined. This is not a limitation since the objective of BCS is to reduce the burden of conducting *in vivo* human studies related to regulatory approval of new formulations of immediate-release products. As presented above, this limitation is also true for BDDCS since DN and the extent of metabolism in humans are required. However, since the primary purpose of BDDCS is to predict drug disposition characteristics, it would be very useful if the BDDCS criteria could be adapted to allow classification of an NME before *in vivo* studies in animals and humans. The observed excellent correlation between the rate of membrane permeability and the extent of metabolism, first recognized by Wu and Benet (1), allows measures of *in vitro* membrane permeability to differentiate BDDCS classes 1 and 2 drugs from BDDCS classes 3 and 4 drugs prior to *in vivo* studies (4). However, as noted above, membrane permeability measurements can be variable, and therefore, the methodology with appropriate standards must be developed in each laboratory carrying out such analyses.

### The Solubility Classification Rule

In 2016, Dave and Morris attempted to define an “early development classification rule” for solubility that could be applied in earlier phases of NME development (10). They reported that by applying a cutoff at 0.3 mg/mL, it was possible to correctly assign BDDCS (and/or BCS) classes to 85% of the drugs for which a solubility value was reported by Wu and Benet (1) at that time (~ 600 drugs). Hence, if the solubility of the NME is above 0.3 mg/mL, it could be assigned to class 1 or 3, whereas if its solubility is below or equal to 0.3 mg/mL, the NME could be assigned to class 2 or 4. Since we have both updated and added new solubility values to the collection, we assessed if the 0.3 mg/mL cutoff is still optimal. Thus, we repeated the analysis done by Dave and Morris and screened cutoffs ranging from 0.1 to 1 mg/mL (i.e., including the 0.3 cutoff) with an increment of 0.01. Not surprisingly, our results show that the 0.3 cutoff retains a remarkable accuracy of 87% (data not shown).

However, we identified a cutoff at 0.44 mg/mL that slightly increases the accuracy to 89% based on our cited solubility data for 1156 drugs. Table VI summarizes the number of correctly and incorrectly classified drugs if the 0.44 mg/mL cutoff had been used before determining the dose number. Correct predictions would have been made for 87.9% of high solubility class 0/1/3 drugs and 91.4% of poor solubility class 2/4 drugs. It is worth noting that all 37 of class 2 or 4 drugs that were incorrectly predicted to be highly soluble are dosed at high quantities (MSD ≥ 150 mg), whereas 64 of 88 of class 1 or 3 drugs that were incorrectly predicted to be poorly soluble are dosed at low quantities (MSD ≤ 10 mg).

**Table VI:** Drugs classified with the updated early solubility classification method

	**Solubility > 0.44 mg/mL**	**Solubility ≤ 0.44 mg/mL**
**BDDCS 0|1|3**	TRUE soluble (637; 87.9%)	FALSE soluble (88; 12.1%)
**BDDCS 2|4**	FALSE insoluble (37; 8.6%)	TRUE insoluble (394; 91.4%)

Therefore, by using a measure of membrane permeability to differentiate classes 1 and 2 from classes 3 and 4 and using the 0.44 mg/mL solubility cutoff to differentiate classes 1 and 3 from classes 2 and 4, it is possible to assign a BDDCS classification to an NME before ever dosing the drug to animals or humans. We estimate that the correct prediction could be obtained for about 85% of small molecule NMEs. We came to this estimate based on the observation of Wu and Benet (1) that most of approved drugs were either EoM ≥ 70% or EoM ≤ 30%, combined with the above analysis that the 0.44 mg/mL cutoff provides accurate solubility prediction for about 90% of approved drugs. This allows drug development scientists to make reasonable predictions concerning the disposition of an NME early in drug development, as detailed below.

## POTENTIAL USES OF BDDCS ASSIGNMENT IN DRUG DEVELOPMENT

### Disposition of Drugs Based on BDDCS Assignment and Potential Modulating Factors to be Considered in Disease States, Drug–Drug Interactions, and Pharmacogenomic Variance

**Fig. 3:**
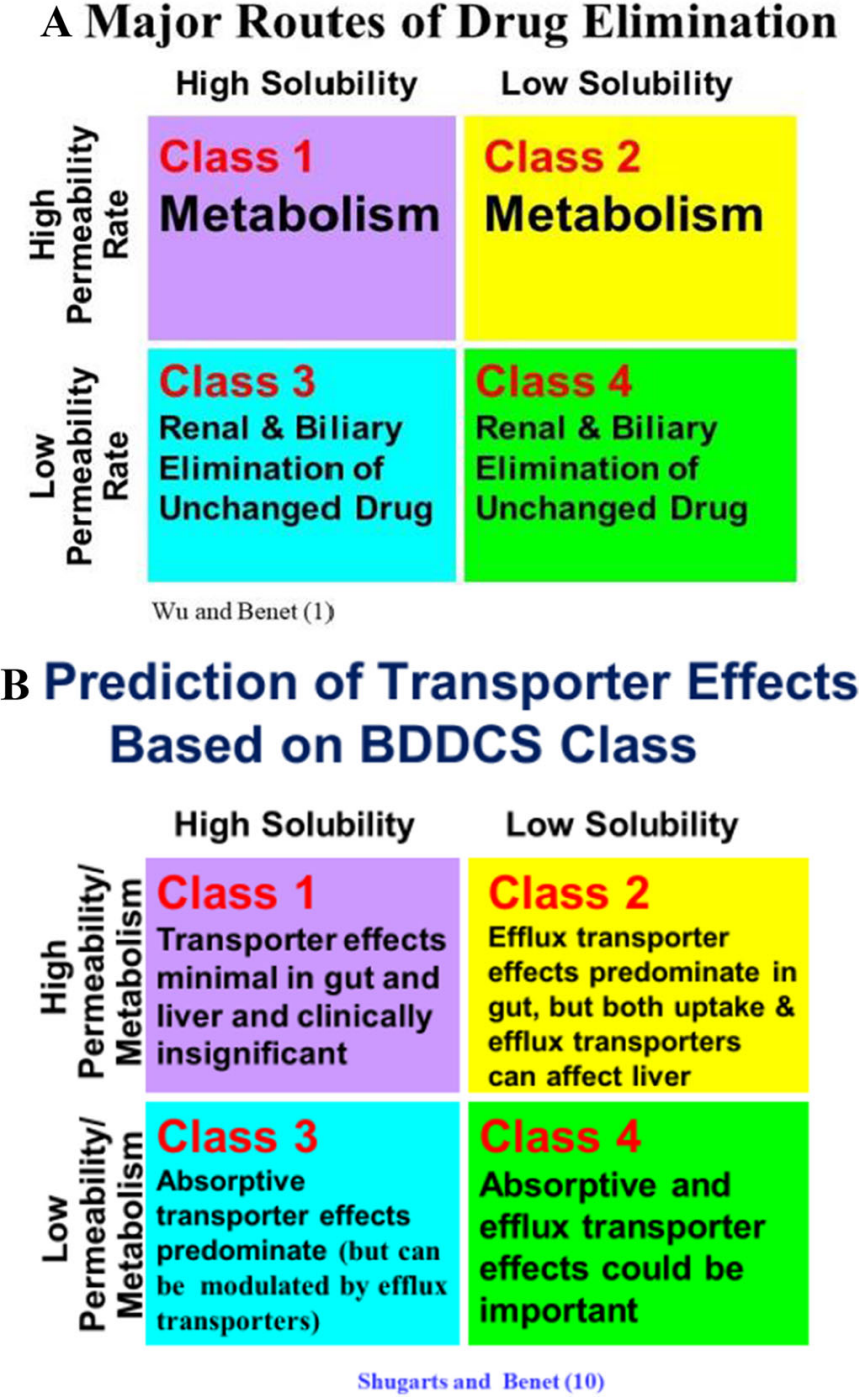
Based on BDDCS: **A** Prediction of major route of drug elimination, **B** Prediction of transporter effects

As depicted in Fig. 3A, from Wu and Benet (1), the predominant route of elimination of BDDCS classes 1 and 2 drugs is via metabolism, both in the liver and intestine, while the predominant route of elimination of BDDCS classes 3 and 4 drugs is via excretion of unchanged drug in the urine or bile. As depicted in Fig. 3B, summarized by Shugarts and Benet (11), even when shown *in vitro* to be transporter substrates, most BDDCS class 1 drugs do not exhibit clinically significant transporter effects in the liver and intestine. In contrast, BDDCS classes 3 and 4 drugs are likely to exhibit clinically significant transporter effects in the liver and intestine because of their poor membrane permeability. BDDCS class 2 drugs, although predominantly eliminated by metabolism, can potentially exhibit both efflux and uptake transporter effects in the liver but only efflux transporter effects in the intestine.

Varma *et al.* (12) expanded the BDDCS findings to provide further predictions of liver and kidney clearance and gut bioavailability through their Extended Clearance Classification System (ECCS), which incorporated differentiation based on substrate molecular weight and charge status. For the ECCS listing of 363 drugs, *in vitro* permeability rate measured by the authors correctly predicted the major route of elimination for 89.5% of the drugs, confirming our conclusion above that *in vitro* permeability measurements provide good prediction of BDDCS classes 1 and 2 drugs *versus* classes 3 and 4 drugs as per Fig. 3A. The major predictions based on ECCS are (a) clearance of high molecular weight (≥400 Da) acids and zwitterions (ECCS class 1B) will be rate-limited by hepatic organic anion transporter polypeptide (OATP) uptake; (b) more recently (13), it is hypothesized that clearance of low molecular weight (<400 Da) acids and zwitterions (ECCS class 1A) may be rate limited by organic anion transporter (OAT) uptake, although the clinical significance of this finding is not confirmed. As predicted by BDDCS the major route of elimination for high permeability ECCS class 1 compounds will be metabolism; (c) acids and zwitterions will not be appreciably metabolized by CYP3A, therefore *F*_*g*_ (fraction of absorbed oral drug unaffected by intestinal metabolism) will be close to 1.0; (d) BDDCS classes 1 and 2 (high permeability) base and neutral compounds (ECCS class 2) will be metabolized in rank order by CYP3A4>UGTs>CYP2D6>esterases,CYP2C; (e) base and neutral high permeability compounds (ECCS class 2) will be preferentially P-glycoprotein (P-gp) substrates affecting *F*_abs_, the fraction of an oral dose that is absorbed; (f) low permeability acids and zwitterions with molecular weight < 400Da will be renally excreted (ECCS class 3A) while those acids and zwitterions with molecular weight ≥400 Da will be rate-limited by OATP uptake, but eliminated predominantly by the renal route; (g) low permeability bases and neutral compounds (ECCS class 4) will be excreted renally.

We concur that the ECCS system is a beneficial addition to BDDCS in predicting drug disposition and bioavailability, and that the addition of criteria related to substrate molecular weight and charge status is important. The addition of the many drugs for which BDDCS has been categorized as presented here, differentiating high from low permeability, should provide a fertile basis for further discoveries related to ECCS or other yet to be identified compound criteria. A major difference between BDDCS and ECCS is providing predictability based on solubility, which is not considered in ECCS, but is a critical determinant in BDDCS and BCS. We expand below on how the solubility criterion is important in predictions of drugs yielding central pharmacodynamics, drug-induced liver injury (DILI), and food effects.

### Improving the Prediction of the Brain Disposition for Drugs Using BDDCS

Broccatelli *et al.* (14) identified 153 drugs that met three criteria: (a) the presence or absence of central human pharmacodynamic effects was known; (b) the drug’s permeability/metabolism and BDDCS class had been assessed; and (c) experimental *in vitro* results were available as to whether the drug was or was not a substrate for P-gp (or ABCB1), since it is generally believed that P-gp substrates do not yield central effects (15). The authors found that 17 of the 153 drugs were high permeability BDDCS class 1 compounds that exhibited significant P-gp efflux *in vitro*. But all 17 of these P-gp substrates, including sertraline, verapamil, and zolmitriptan, exhibit central pharmacodynamic effects. This supports the conclusion for BDDCS class 1 drugs presented in Fig. 3B that transporters are clinically insignificant, and that this also holds for other membranes, including the brain. To make such an assessment on the potential for blood-brain barrier permeability, the differentiation among high permeability compounds requires knowledge of a drug’s solubility. The important implication of these results in drug development is that BDDCS class 1 compounds are likely to be brain permeable and achieve pharmacodynamically relevant concentrations, whether this is desired or not. This could be a strong rationale for not always wanting a class 1 NME. We have recently shown that almost all antidepressants (16) and antihypertensives (17) are BDDCS class 1 drugs.

### Using BDDCS to Validate the Usefulness of DILI Predictive Metrics

**Fig. 4:**
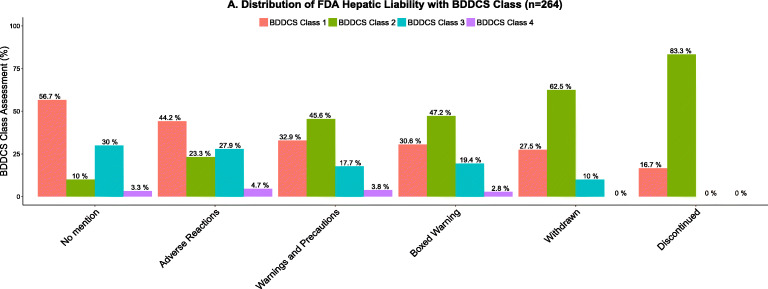
Distribution by BDDCS class of hepatic liability for FDA listing of 264 drugs as reported by Chan and Benet (16)

DILI is the leading cause of drug failure in clinical trials and a major reason for drug withdrawals from the market. Idiosyncratic DILI is very complex: several mechanisms appear to induce an immune response, reactive metabolites appear to be involved in most idiosyncratic DILI, and DILI is dependent on both dose and extent of hepatic metabolism. Many toxicology efforts are dedicated to developing methodologies to predict DILI for an NME that are complex and time-consuming. However, we have found that these methodologies often do no better than just avoiding BDDCS class 2 compounds (18, 19). As seen in Fig. 4, with increasingly severe indicators of hepatic liability, more and more drugs fall into BDDCS class 2. In our analysis, none of the DILI predictive metrics, except keeping daily dose < 50 mg, provides any better prediction of DILI than just avoiding BDDCS class 2 drugs.

The advantage of the BDDCS system is that the BDDCS class prediction can be made before ever knowing the daily dose. However, many valuable BDDCS class 2 drugs do not cause DILI. Our papers (18, 19) explicitly state that BDDCS classification should not be used as a DILI predictive metric. But we emphasize that if a new DILI predictive metric cannot be differentiated from BDDCS class 2, there can be no confidence in the metric and the toxicity hypotheses implied. Toxicologists are not familiar with BDDCS or BCS and generally ignore our recommendations, spending considerable resources developing metrics that most often cannot be differentiated from this simple caution of avoiding BDDCS class 2 drugs. However recently, Brecklinghaus *et al.* (20), summarizing the collaborative effort of several academic and industry European and Mid-East toxicology units, recognized these observations writing: “In future, it will be important to study if readouts from in vitro tests e.g., cytotoxicity, carrier inhibition, gene expression alterations, reactive metabolite formation etc. will improve DILI prediction independent from BDDCS class. For this purpose, large sets of compounds (>100) with sufficient substances from all four BDDCS will be required.”

### Predicting Food Effects Using BDDCS Prior to *In Vivo* Studies in Animals or Humans

**Fig. 5:**
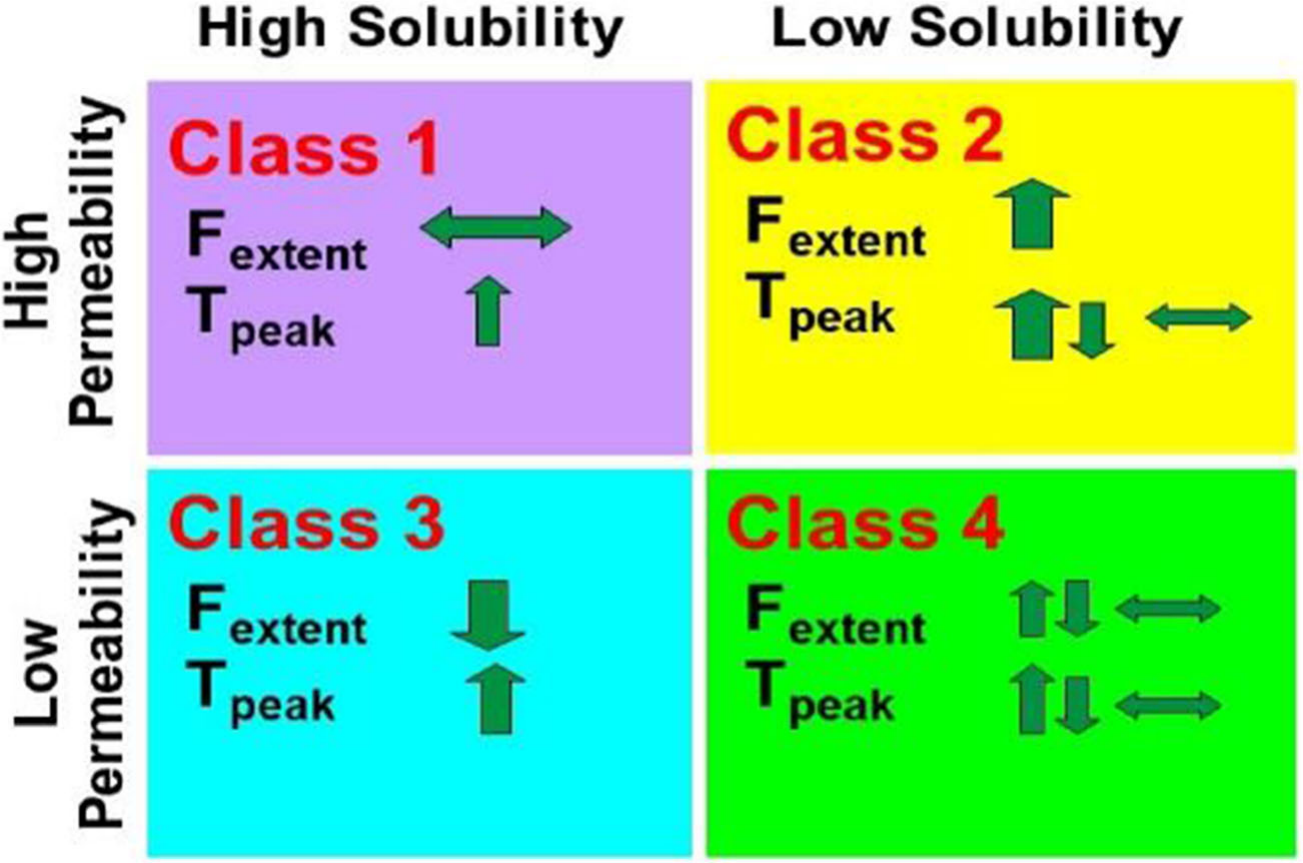
Summary of the effects of high fat meals on the extent of bioavailability (*F*_extent_) and peak time (*T*_peak_) for BCS class drugs as presented by Fleisher *et al.* (20) adapted from Custodio *et al.* (7)

All approved drug products must be studied to determine the effects of high-fat meals on the bioavailability of the proposed dosage form, and this information is included in the drug label (21). In 1999, Fleisher *et al.* (22) summarized published studies examining the effects of high-fat meals on various BCS classified drugs as summarized in Fig. 5 adapted from Custodio *et al.* (7). Meals generally slow down stomach emptying causing the peak time (*T*_peak_) to increase with the highly soluble classes 1 and 3 compounds and most class 2 compounds. There were too few class 4 compounds to come to any conclusion. However, the extent of bioavailability (*F*_extent_) exhibited differences between class 1 drugs (where little change is observed), class 2 drugs where bioavailability is generally increased with a high-fat meal, and class 3 compounds where bioavailability is generally decreased. It is difficult to rationalize these findings as food effects and drug absorption are complicated processes. One might argue that high-fat meals would increase the intestinal concentrations of poorly soluble but highly permeable class 2 compounds and decrease the intestinal concentrations of highly soluble poorly permeable class 3 compounds, but why is no effect seen with highly soluble, highly permeable class 1 compounds? Custodio *et al.* (7) speculated that the outcomes were consistent with high-fat meals inhibiting intestinal efflux transporters, but we conclude that the outcome only appears to be predictive for about 70% of food effect studies. Recently, there has been interest in the ability of physiologically based pharmacokinetic (PBPK) modeling to predict food effects quantitatively, but the outcomes have not provided sufficient validation as reviewed in an FDA-authored publication (23). Most recently, Wagner *et al.* (24) examined the potential reasons for poor PBPK food effects predictions for two BDDCS class 2 drugs exhibiting increased *F*_extent_ (pazopanib and ziprasidone) and a BDDCS class 3 drug exhibiting decreased *F*_extent_ (trospium). Notice that these directional changes would have been correctly predicted following Fig. 5. The 2019 FDA-authored study (23) examined predictability for 39 drugs, but only 8 were identified. BDDCS and Fig. 5 would have predicted the direction of change correctly for 7 of the 8 (erring on nifedipine, a BDDCS class 2 drug showing no significant change). We note that two of the drugs, ceritinib (designated BCS class 4) and cinnarizine (designated BCS class 2/4), are highly metabolized and BDDCS class 2 drugs with food effects causing increased *F*_extent_ as per Fig. 5. We believe it is important to use BDDCS rather than BCS classification in evaluating these retrospective data due to the uncertainty of the *in vitro* permeability measures and the fact that BCS is based on permeability extent rather than permeability rate, where the latter is a better predictor of extent of metabolism. We still believe that there is not a sufficient number of BDDCS class 4 drugs studied to make any solid prediction, but our suggestion is increased *F*_extent_. Predicting the presence of and the direction of food effects using BDDCS before an NME has been dosed to either animals or humans is a useful tool in preclinical drug development. BDDCS predictions are better than any animal food effect studies, and we recommend such animal studies should not be carried out. The field is a long way from predicting food effects quantitatively using PBPK approaches, and we recommend that regulatory agencies continue to require such studies in humans.

## RECOMMENDATION FOR BDDCS AND ECCS ASSIGNMENT EARLY IN DRUG DEVELOPMENT

To differentiate BDDCS and ECCS classes 1 and 2 drugs from classes 3 and 4 drugs prior to dosing of an NME to animals and humans, it is necessary to have a reliable rate of permeability assay method that correctly differentiates a reasonably large set (≥ 20) of approved drug formulations with known drug BDDCS and ECCS assignment. Then ECCS could be used to predict drug disposition class via molecular weight and charge. The almost 1500 drugs for which permeability is classified here can serve as the basis for further compound criteria discoveries beyond ECCS. With a 0.44-mg/mL water solubility cutoff, BDDCS assignments could inform further ECCS predictions, followed by additional predictions related to brain penetration, DILI potential, and food effects.

## CONCLUSIONS

In this work, we have provided new BDDCS classification for 379 drugs, and we have described revisions for drugs that were already classified with BDCCS. We detail revised class assignment of previously misclassified drugs and references for the classification of new and previously classified drugs for maximum approved dose, extent of excretion of available drug excreted unchanged in the urine, and lowest solubility over the pH range 1.0–6.8, when such information is available. We compare BDDCS and BCS classification for 257 BCS categorized drugs. We update the early development classification rule by increasing the solubility threshold from the original 0.3 mg/mL to the slightly more accurate 0.44 mg/mL. We detail the uses of ECCS and BDDCS in predicting drug disposition characteristics prior to dosing animals or humans, the use of BDDCS to predict potential brain penetration, the outcome of food effect studies, and drug-induced liver injury (DILI) potential. This work provides an update on the current status of the BDDCS and its uses in the drug development process.

## Key to Utilizing the Supplementary Information

All data associated with this work is available in Supporting information Tables S1–4. Table S1 lists information for the BDDCS classified compounds: drug name, synonyms, CAS #, year of approval, PubChem ID, SMILES, InChI, and charged state. Table S2 reports the current BDDCS assignment, and the parameters used to generate it, separated by collection: Benet et al. (5; as LZB2011), Hosey et al. (6; CMH2016), and the present additions (GB2021). In Table S3, the detailed revision of the data is reported. In the case of revisited drugs, both the former and the updated values are listed for fraction excreted unchanged in urine, maximum dose strength, solubility, dose number, and BDDCS assignment. For newly classified drugs, the new values only are reported in the columns labelled with [UPDATED]. If detected during the review process, the fraction of drug excreted unchanged in the bile is also reported. The drug transformation (i.e., whether the compound is a prodrug or an active metabolite) and the route of administration are also saved in this table. Additionally, in Table S3, metabolism and solubility data are assigned to a unique reference ID to provide an easy way to access the original data source. These IDs are listed in Table S4 along with the link to the original paper, drug label, etc. Table S5 lists the current BCS information for drugs. BCS classes were collected mainly from three publications: Lindenberg et al. (25), Wu and Benet (1), Box and Comer (26). When new BCS data were found from the inspection of FDA or EMA documents, we recorded and listed it as well (see GB2021 in Table S5). Finally, to facilitate the merging of the data across Tables S1‐3 and 5, a unique ID (BDDCS.ID) is assigned to each compound in the collection and can be found in Tables S1, S2, S3, and S5.

## Supplementary Information

ESM 1 (XLSX 537 kb)

## Abbreviations

*BCS*: Biopharmaceutics Classification System
*BDDCS*: Biopharmaceutics Drug Disposition Classification System
*DILI*: Drug-induced liver injury
*DN*: Dose number
*ECCS*: Extended clearance classification system
*EoM*: Extent of metabolism
*F*_*abs*_: Fraction of an oral dose that is absorbed through the wall of the gastrointestinal tract
*f*_*e*_: Fraction of the systemically available dose that is excreted unchanged in urine
*F*_*extent*_: Fraction of an oral dose that reaches the systemic circulation
*F*_*g*_: Fraction of the absorbed oral dose that is not metabolized in the intestine
*MDS*: Approved maximum dose strength
*NME*: New molecular entity
*OAT*: Organic anion transporter
*OATP*: Organic anion transporter polypeptide
*PAMPA*: Parallel artificial membrane permeability assay
*PBPK*: Physiologically based pharmacokinetic
*P-gp*: P-glycoprotein
*SOL*_*FDA*_: The lowest drug solubility measured across the pH range 1–6.8
*T*_*peak*_: Time of peak concentration following an oral dose
